# Highly Parallel Genomic Selection Response in Replicated *Drosophila melanogaster* Populations with Reduced Genetic Variation

**DOI:** 10.1093/gbe/evab239

**Published:** 2021-10-25

**Authors:** Claire Burny, Viola Nolte, Marlies Dolezal, Christian Schlötterer

**Affiliations:** 1 Institut für Populationsgenetik, Vetmeduni Vienna, Austria; 2 Vienna Graduate School of Population Genetics, Vetmeduni Vienna, Austria; 3 Plattform Bioinformatik und Biostatistik, Vetmeduni Vienna, Wien, Austria

**Keywords:** experimental evolution, evolve and resequence, inbred strains, polygenic trait, parallelism, *Drosophila melanogaster*

## Abstract

Many adaptive traits are polygenic and frequently more loci contributing to the phenotype are segregating than needed to express the phenotypic optimum. Experimental evolution with replicated populations adapting to a new controlled environment provides a powerful approach to study polygenic adaptation. Because genetic redundancy often results in nonparallel selection responses among replicates, we propose a modified evolve and resequence (E&R) design that maximizes the similarity among replicates. Rather than starting from many founders, we only use two inbred *Drosophila melanogaster* strains and expose them to a very extreme, hot temperature environment (29 °C). After 20 generations, we detect many genomic regions with a strong, highly parallel selection response in 10 evolved replicates. The X chromosome has a more pronounced selection response than the autosomes, which may be attributed to dominance effects. Furthermore, we find that the median selection coefficient for all chromosomes is higher in our two-genotype experiment than in classic E&R studies. Because two random genomes harbor sufficient variation for adaptive responses, we propose that this approach is particularly well-suited for the analysis of polygenic adaptation.

Significance

Adaptation to temperature has a polygenic basis, which makes the molecular characterization challenging. Either the contribution of individual loci is too small to be detected experimentally or the contributing loci are genetically redundant, which implies that the same phenotype can be obtained by different allelic combinations. Here, we propose an experimental framework, which uses only two founder genotypes to obtain a strong and highly parallel selection response to a new, hot temperature regime. We suggest that such parallel selection responses in replicated populations provide an excellent opportunity to characterize polygenic adaptation, because it avoids uncertainty about selection signatures typically associated with genetic redundancy.

## Introduction

 Many adaptive traits have a polygenic basis (Hoffmann et al. 2003; Barton and Etheridge 2018; Barghi et al. 2020), where typically more contributing loci are segregating in a population than needed to reach the trait optimum (Yeaman 2015). For highly polygenic traits, the contribution of a single locus during adaptation to a new environment, that is, a new phenotypic optimum, will be small, usually too small to be detected by classic population genetic tests (Pritchard et al. 2010; Pritchard and Di Rienzo 2010). Thus, tests for polygenic adaptation aggregate signals across multiple loci to gain statistical power (Turchin et al. 2012; Berg and Coop 2014; Sella and Barton 2019). However, distinguishing the contributions of demography and selection in these aggregated signals can be challenging in natural populations because of residual population structure (Barton et al. 2019; Berg et al. 2019; Sohail et al. 2019). Hence, experimental evolution has been proposed as an alternative approach to study polygenic adaptation (Vlachos and Kofler 2019; Barghi et al. 2020; Lou et al. 2020). Laboratory natural selection within the evolve and resequencing (E&R) framework (Garland and Rose 2009; Turner et al. 2011; Long et al. 2015; Schlötterer et al. 2015) has been successfully used to study adaptation in controlled environments, combining experimental evolution and Pool-Sequencing (Pool-Seq) on replicated populations (Schlötterer et al. 2014).

Simulation studies (Baldwin-Brown et al. 2014; Kofler and Schlötterer 2014; Kessner and Novembre 2015) recommend optimizing different design parameters to obtain a good mapping resolution. An established strategy is to use a large number of founder genotypes. Maximizing the number of founders provides the advantage that contributing alleles segregating at intermediate frequency will be located on multiple haplotypes, which facilitates their identification (e.g., Kofler and Schlötterer 2014). This experimental design is well-suited for the selective sweep paradigm without epistasis for fitness—a hallmark of polygenic adaptation. For highly polygenic traits, increasing the number of founders also increases the number of available contributing alleles, which may either trigger competition between segregating haplotypes if they interfere with each other (Hill and Robertson 1968), or inflate genotypic redundancy making evolution less repeatable (Láruson et al. 2020). Additionally, increasing the number of founders lowers the starting frequency of haplotypes, which in turn increases their chance to be lost by drift—also resulting in lower parallelism.

As a consequence, a (highly) heterogeneous response between replicates is expected for highly polymorphic founder populations and has been seen in several E&R studies (e.g., Griffin et al. 2017; Hardy et al. 2018; Seabra et al. 2018; Barghi et al. 2019; Rêgo et al. 2019)—even when the same founder population is used. In small populations where stochastic sampling effects have a strong impact on allele frequencies, this may either increase or decrease the frequency of contributing alleles during the early generations and thus result in heterogeneous selection responses across replicates (Bolnick et al. 2018; Nené et al. 2018; Langmüller and Schlötterer 2020). Although this genetic redundancy provides convincing evidence for polygenic architecture, it is challenging to distinguish nonparallel selection responses from genetic drift. We showed recently that a follow-up E&R study (secondary E&R) can confirm selection signatures, which are restricted to a single replicate (Burny et al. 2020), but the large number of segregating haplotypes results in very complex allele frequency changes (AFCs) preventing a further characterization of the selection target (Langmüller et al. 2021).

Given these challenges to characterize the adaptive response with a classic E&R design based on a large number of founder genotypes, we propose an alternative design. Using only two different genotypes is the most dramatic reduction of variation segregating in natural populations, which offers the potential to distinguish selection signatures from stochastic changes based on highly parallel selection responses. Given the success of bulk segregant analysis based on Pool-Seq of individuals with extreme phenotypes—Ehrenreich et al. (2010) identified up to 20 contributing loci from a cross of two yeast genotypes, we reasoned that a two-genotype E&R study may provide a powerful approach to study the adaptive architecture. Unlike the genetic architecture which also includes loci that cannot respond to selection due to deleterious pleiotropic effects, the adaptive architecture focuses on variants contributing to adaptation (Barghi et al. 2020).

A two-genotype E&R study provides the conceptual advantage that much less standing genetic variation is available to contribute to adaptation and all contributing alleles will start from the same frequency. The selection response should be less complex and more parallel across replicates compared with classic E&R studies. Similar to classic E&R studies, selection targets can be identified by a frequency increase during experimental evolution. The major conceptual difference is, however, that the selection targets are located only on two homologous chromosomes, which implies that the favorable alleles located on the same chromosome can only be detected in two-genotype E&R when they are separated by a nonfavored allele. Recombination during the experiment will uncouple the nonfavored allele, resulting in a selection signature that distinguishes three selection targets. Nevertheless, the ability to distinguish these selection targets depends on multiple factors: effect size, recombination rate, distance between loci as well as the extent of the parallel selection response between replicate populations. Because the degree of parallelism is the only parameter that could be (indirectly) modified by the experimental design, it is of key importance to know if typically used population sizes are resulting in a selection signature, which is sufficiently parallel to distinguish noise (drift and sampling of reads in Pool-Seq) from the biological signal.

In this study, we explore the potential of two-genotype E&R as an alternative approach to characterize the genetic architecture of a temperature adaptation. Although a putatively polygenic trait (Angilletta 2009; Barghi et al. 2019; Dayan et al. 2019; Herrmann and Yampolsky 2021), chill coma recovery time, a measure of adaptation to cold temperature, has a very simple genetic basis in Drosophila *ananassae* (Königer et al. 2019). Thus, the adaptive architecture for temperature may range from a small number of loci of large effect to a highly complex one with many loci, each with very small effects—close to the infinitesimal model.

**Fig. 1.**
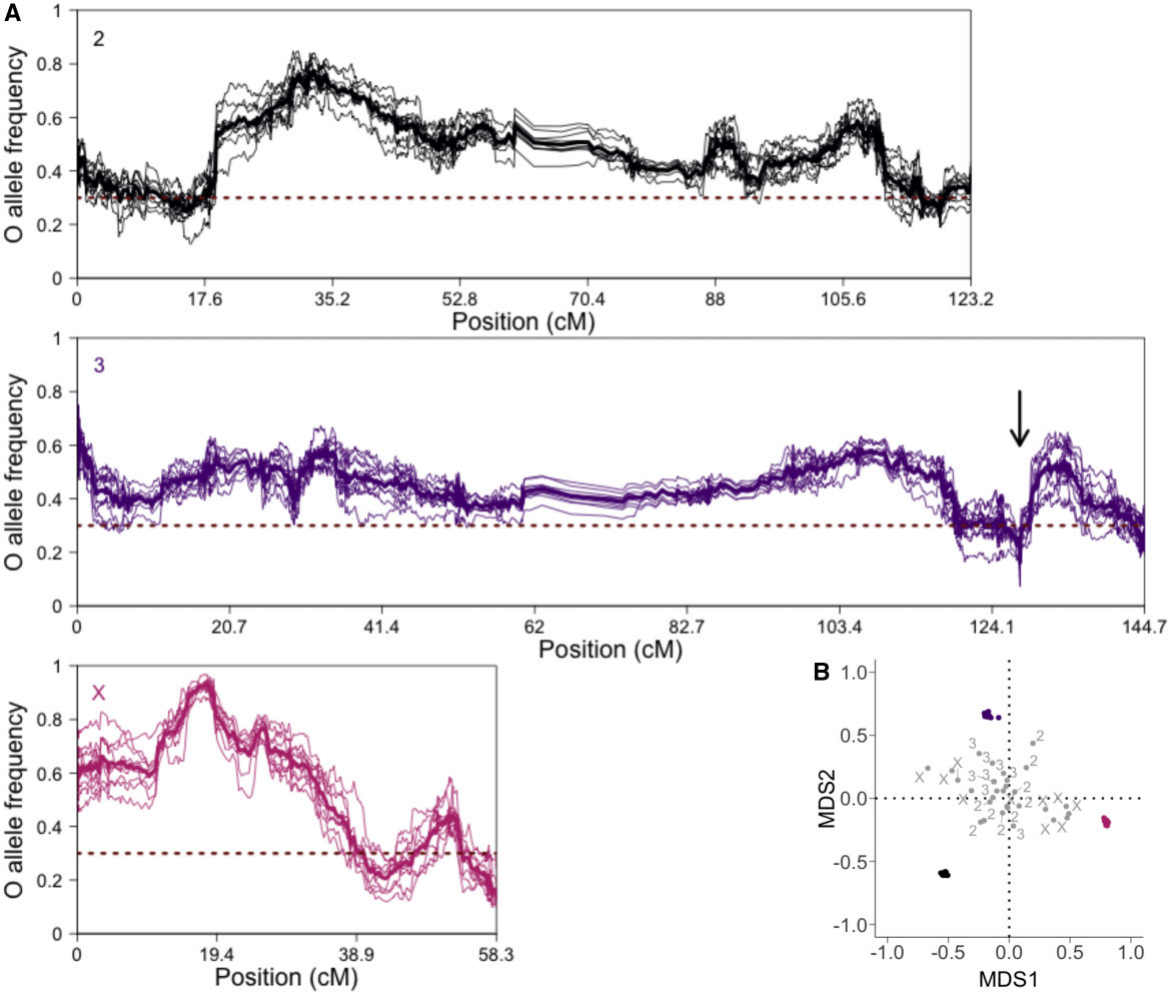
Strong parallel response after 20 generations of evolution at 29 °C. (*A*) Smoothed Oregon-R (O) AF (*y* axis) at F20 in all replicates colored by major chromosome in cM unit (*x* axis). The same color code applies to all figures (pink: X; black: 2; purple: 3). The mean O AF is computed over nonoverlapping windows of 250 SNPs. The bold line represents the mean O AF per window over the 10 replicates and the horizontal dotted line the starting O AF (0.3). The arrow indicates the position of a localized selection signature on chromosome 3 (see Discussion). (*B*) 2D MDS projection of the pairwise *ρ* Spearman correlation matrix between empirical (colored) and neutral (gray) allele frequencies per major chromosome. The correlation coefficient values were transformed to distances (2√(1 − *ρ*)) prior to projection.

We studied the parallel selection response from two founder haplotypes by creating 10 replicate populations from two parental inbred *Drosophilamelanogaster* strains, Samarkand and Oregon-R, which were exposed to an extreme temperature regime (constant 29 °C). Because this temperature is only slightly below the maximum temperature at which *D. melanogaster* are viable and fertile (fig. 1, Hoffmann 2010), we maximize the chance of a parallel selection response. Eventually, all contributing alleles that start at intermediate frequency in the founder population will be measured after 20 generations. Using this design we asked two questions:

1. Is temperature sufficiently polygenic to find selection signatures on multiple chromosome arms?
2. Is it possible to create sufficiently parallel selection responses, which can distinguish between selection and stochastic forces—even for moderate selection effect?

By analyzing the genomic responses in the 10 replicate populations maintained for 20 generations at a hot temperature, we find that two founder genotypes harbor enough natural variation to ensure a selective response. A very strong and highly parallel selection signature is seen in all replicates, which enabled us to recognize linked alleles of opposite effects even when the resulting AFCs were moderate. This demonstrates that even for temperature adaptation, which is highly polygenic, an adequate experimental design, that is, a reduced founder diversity and a distant trait optimum, results in reproducible selection signals.

## Materials and Methods

### Experimental Setup

We used the Oregon-R and Samarkand strains inbred by Chen et al. (2015), and maintained since then at room temperature. The experiment started with 10 replicates, each with a census size of 1,500 flies and accidentally with a starting frequency of 0.3 for the Oregon-R genotype (0.7 for the Samarkand genotype)—rather than 0.5 of each genotype. The ten replicates were then maintained in parallel at a constant 29 °C temperature in dark conditions. Every generation, 300 adults were transferred to one of five bottles for 2 days of egg laying. After egg laying, all adults were removed and frozen. Because the egg lay resulted in a high density of larvae, a mixture of larvae and food was transferred to two fresh food bottles. Adults were collected 8–32 h after the first flies eclosed. Adults from all bottles were mixed to avoid population substructure and 300 adults from each vial gave rise to the next generation.

### DNA Extraction, Library Preparation, and Sequencing

Whole-genome sequence data for the parental Oregon-R and Samarkand strains are available from Chen et al. (2015). The ten evolved replicates in generation F20 were sequenced using Pool-Seq: genomic DNA was prepared after pooling and homogenizing all available individuals of a given replicate in extraction buffer, followed by a standard high-salt extraction protocol (Miller et al. 1988). Barcoded libraries with a targeted insert size of 480 bp were prepared using the NEBNext Ultra II DNA Library Prep Kit (E7645L, New England Biolabs, Ipswich, MA) and sequenced on a HiSeq 2500 using a 2 × 125-bp paired-end protocol.

### Establishment of a Parental SNPs Catalogue

After quality control with FastQC (http://www.bioinformatics.babraham.ac.uk/projects/fastqc/; last accessed January 2021), the raw reads were demultiplexed and trimmed using ReadTools (Gómez-Sánchez and Schlötterer 2018; version 1.5.2; *–mottQualityThreshold 18, –minReadLength 50, –disable5pTrim true*) leading to a mean insert size of 392 bp. The processed paired-end reads were mapped using NovoAlign (http://novocraft.com; last accessed April 2021; version 3.09; *-i 250,75 -F STDFQ -r RANDOM*) on the combined *D. melanogaster* reference genome v6.03 (Thurmond et al. 2019), *w*Mel (AE017196.1), *w*Ri (CP0013391.1), and common gut bacteria (*Acetobacter pasteurianus* [AP011170.1]; *Lactobacillus brevis* [CP000416.1]). From the processed BAM files, that is, without duplicates (using PICARD MarkDuplicates; http://broadinstitute.github.io/picard/; last accessed April 2021; version 2.21.6; *REMOVE_DUPLICATES=true VALIDATION_STRINGENCY=SILENT*), quality filtered (using samtools [Li et al. 2009]; version 1.10; *-b -q 20 -f 0x002 -F 0x004 -F 0x008*) and reheadered. The average coverages are 60 and 62× for Samarkand and Oregon-R strains and 146, 129, 158, 150, 159, 142, 186, 117, 127, 140× for replicates 1 to 10, respectively. Multisample variant calling using the two processed parental BAM files was done with Freebayes (Garrison and Marth 2012; version 1.3.5). Biallelic SNPs in regions outside **≥**200 bp length repeats (identified by RepeatMasker, http://www.repeatmasker.org; last accessed October 2020) were retained using vcftools (Danecek et al. 2011; version 0.1.16; *–min-alleles 2 –max-alleles 2*) and bedtools (Quinlan and Hall 2010; version 2.27.1; *intersect*). The vt toolbox (Tan et al. 2015) was used to normalize and to decompose variants (version v0.57721; *normalize*; *decompose_blocksub; uniq*). We removed SNPs within 5 bp of an indel using bcftools (Li 2011; version 1.9; *filter -g5 -i ‘TYPE=“snp”’*) (soft filters). The filtered VCF was loaded in R using the *vcfR::read.vcfR* R function (Knaus and Grünwald 2017; version 1.12.0). We retained SNPs covered in both parents (based on the sum of AO and RO) within the averaged 1st–99th percentile coverage (11–68). We eventually kept SNPs with a QUAL value greater than 413 (1st percentile of QUAL values **≥**1), leading to a total of 684,065 processed SNPs (hard filters). A parental SNP was defined as a (nearly) fixed difference between parental lines with a 0/0 (1/1) genotype in the Samarkand parent and 1/1 (0/0) genotype in the Oregon-R parent at the marker position, conditioning for a frequency of the alternate allele lower than 0.05 (if 0/0) or higher than 0.95 (if 1/1). We obtained a final list of 465,070 SNPs (see supplementary table S1, Supplementary Material online for a detailed count of markers at each filtering step); 401,252 and 63,818 SNPs on the autosomes and the X chromosome, respectively, equivalent to 1 SNP every 271 bp on the autosomes and 363 bp on X. Allele frequencies at these informative parental marker SNPs are obtained after converting processed F20 BAM files from pileup (*samtools mpileup -BQ0 -d10000*) to sync files using PoPoolation2 (Kofler et al. 2011; *mpileup2sync.jar*). The subsequent analyses have been performed with R (version 4.0.4; R Core Team 2018) and most figures have been done with the ggplot2 R package (Wickham 2016). For the parental strains, we used the frequency of inversion-diagnostic SNPs to check the inversion status of common cosmopolitan inversions as inversions would impede recombination (Kapun et al. 2014). Both parental strains are homosequential (supplementary fig. S1, Supplementary Material online). We also checked the density of heterozygous SNPs per parent prior to QUAL filtering and obtained 2,711 and 2,698 heterozygous SNPs (genotyped 0/1) in Samarkand and Oregon-R, respectively, representing less than 0.6% of the total number of parental marker SNPs.

### Allele Frequency Tracking

At each SNP, we obtained counts for both parental alleles from the F20 sync files. We polarized allele frequency (AF) for the Oregon-R allele. The frequency of the Samarkand allele is obtained as 1 minus the Oregon-R AF. The AFC of a given marker is signed; if the Oregon-R AF at F20 is higher (lower) than 30%, the Oregon-R (Samarkand) allele increased in frequency and the AFC is positive (negative). The genome was partitioned in 1,862 nonoverlapping genomic windows of 250 parental SNPs; 1,606 on the autosomes, 256 on the X chromosome, spanning on average 67.8 and 90.5 kb on the autosomes and X. Note that the last windows of chromosomes 2, 3, and X contain 20, 160, and 68 SNPs, respectively and are not included in the analysis. The AF per window was summarized as the mean over 250 SNPs. A window position *i* is defined by its center ((right bound − left bound)/2). Markers along the genome are positioned in cM unit, to adjust for heterogeneity in recombination rate along the chromosome. The recombination map of Comeron et al. (2012) was updated to version 6 of the reference genome using the Flybase online Converter (https://flybase.org/convert/coordinates; accessed in July 2020). Physical chromosome positions were converted to genetic positions via interpolation (Gatti et al. 2014; version 1.0.0; *DOQTL::fill.in.snps* R function) to avoid SNPs that have the same cM value to overlap at the cM scale (cf Marey map in supplementary fig. S2, Supplementary Material online, Mb unit in supplementary fig. S3, Supplementary Material online). The effective population size, *N_e_*, was estimated per replicate for the autosomes and X separately using the *poolSeq::estimateNe* R function (Taus et al. 2017) from 10,000 randomly picked SNPs and summarized as the median over 1,000 trials, similarly as in Vlachos et al. (2019) (supplementary table S2, Supplementary Material online).

### Quantification of the Response

For each replicate, we reported the median AF of the Oregon-R allele over the windows. We also reported the median coefficient of variation (CV) per chromosome to quantify the deviation around the average AF value per window. We additionally computed the autocorrelation in AF between windows using the *acf* R function. ACF at a given step *k* is defined as the correlation between windows at positions *i* and *i + k*, where *k* is called the lag. We used the median distance in Mb at which a significant decrease in ACF was noted (*α* = 5%, below 1.96/*√m*, *m* the number of windows) as a rough proxy for linkage disequilibrium (LD).

We performed neutral simulations mimicking our empirical design (starting frequency of 0.3 for the Oregon-R alleles, 10 replicates, 20 generations, unbiased sex ratio, census size of 1,500 flies) using MimicrEE2 (Vlachos and Kofler 2018). From the simulated sync files, we then drew the coverage per SNP from a Poisson distribution (mean = 122 reads, estimated from the empirical read counts) and performed binomial sampling with the sample size equal to the coverage as suggested in Taus et al. (2017), to reproduce Pool-seq sampling noise. To contrast our empirical results with neutral expectations, we computed the pairwise *ρ* Spearman correlation matrix between all neutral and empirical replicates (10 replicates times 2) per arm (3 major chromosomes), leading to a 10 × 2 × 3 entry matrix. The *ρ* values were converted to distances (2√(1 − *ρ*)) prior to projecting the distance matrix in two dimensions with multidimensional scaling (MDS; Gower 1966). The significance of the pairwise correlations was assessed with *t*-tests separately for empirical and neutral replicates, where *P* values were adjusted with a Benjamini–Hochberg correction. We performed a sign test for the median AFC to test if the median AFC per major chromosome is higher than 0, where *P* values were adjusted with a Benjamini–Hochberg correction.

We validated the selection signatures visually identified on the X chromosome by simulations with MimicrEE2 (Vlachos and Kofler 2018), starting with the same haplotype file used for the neutral simulations, and the *D. melanogaster* recombination map (Comeron et al. 2012). Key to our simulations was that we iteratively increased the number of targets (1, 2, 3, 4, 6) to improve the fit of the simulated Oregon-R frequencies to the empirical frequencies. The selection targets were defined as follows: we picked a random SNP within windows that are local extrema of the average selection coefficients *s* along the X chromosome, detected with the *ggpmisc::find_peaks* R function (Aphalo 2021; version 0.4.3). If the observed *s* value at the SNP is positive (negative), the beneficial allele is an Oregon-R (Samarkand) allele. The selection coefficient values were iteratively adjusted to improve the fit to the empirical frequencies. The simulations started with the most strongly selected SNP followed by the strongest responsive site of opposite effect—this resulted in three SNPs with positive effect and three SNPs with negative effect. The final *s* values are indicated in supplementary fig. S4, Supplementary Material online. The goodness of fit for each the five simulation sets (1, 2, 3, 4, 6 targets) to the average smoothed empirical allele frequencies was measured by the sum of squared estimate (SSE) of error. We also performed paired *t*-tests to compare the average smoothed empirical allele frequencies to the simulated ones for each of the five scenarios, where *P* values were adjusted with a Benjamini–Hochberg correction.

### Comparisons to Other Data Sets

We qualitatively contrasted our study with two additional E&R studies (table 1) that are similar in terms of duration and lack inversions but start with hundreds of founder genotypes, and thus heterogeneous starting allele frequencies. To compare studies, we computed the absolute selection coefficient per SNP in all available replicates; ten replicates in this study, three replicates from Kelly and Hughes (2019) (between F0 and F15) and ten replicates from Barghi et al. (2019) (between F0 and F20) using the same number of SNPs (10,000) for each study and for each chromosome X, 2, and 3. To avoid problems with SNPs fixed in one or more populations, we first generated pseudocounts by subtracting (adding) a pseudocount of 1 to fixed (lost) SNPs, as described in Vlachos et al. (2019). *N_e_* was estimated as described above. We then estimated the selection coefficient *s* of each biallelic marker SNP, assuming independence of selection targets (no linkage and independent effects on fitness) and codominance (*h* = 0.5), using the *poolSeq::estimateSH* R function (Taus et al. 2017) by fitting a linear model with least square regression adjusted for bias on the logit-transformed Oregon-R allele frequencies; $\ln(\frac{AF(t)}{1-AF(t)})=\ln(\frac{AF(t0)}{1-AF(t0)})+\frac{t\;s}{2}$ with *t* time in generations. We determined the 95% *t*-based confidence interval of *s* for each replicate and major chromosome by jackknifing using the *bootstrap::jackknife* R function (Efron and Tibshirani 1994; Leisch et al. 2019; version 2019.6).

**Table 1.** E&R Data Sets Information

Number of Founder Genotypes	Census Size	Pressure	Species	Generation Used	Sequencing Information	Publication
2	1,500	LNS constant 29 °C	*D. melanogaster*	20 non-overlapping	Pool-Seq of 1,500 mixed males and females	This study
**≈**800	1,000	LNS fluctuating temperature (28 °C/18 °C, mean 23 °C)	*D. simulans*	20 non-overlapping	Pool-Seq of 1,000 mixed males and females	Barghi et al. (2019)
**≈**1,000	Mean = 1,277; Range = 832–1,635 Mean = 849; Range =672–1,147 Mean = 1,187; Range = 963–1,620; for replicates A, B, C	LNS constant temperature (25 °C)	*D. simulans*	15 overlapping	Pool-Seq of 500 males and females	Kelly and Hughes (2019)

## Results

### Parallel Response after 20 Generations of Evolution at High Temperature

Using two genotypes to set up the founder population provides the advantage that all parental alleles start from the same frequency across the entire genome. A simple genome-wide FC plot along the genome provides an intuitive visualization of the selection targets (fig. 1*A*): the pronounced FC increase of the putatively selected alleles, either Oregon-R (AF > 30%) or Samarkand (AF < 30%), generates a “hill-valley-like” landscape. Because recombination rate a priori determines the width of the genomic region affected by a selected site (Felsenstein 1974; Barton 1995; Otto and Lenormand 2002; Roze and Barton 2006), we scaled the chromosomes in cM unit (for a base-pair scaling, see supplementary fig. S3, Supplementary Material online). Throughout the entire genome, we observe a fast and strong response after 20 generations (fig. 1*A*) where in all replicates, large, linked genomic regions experience very similar changes in frequency.

The high level of parallelism among the empirical replicates is reflected in highly correlated allele frequencies between replicates, higher than 0.8 (*t*-test on pairwise Spearman correlation coefficient *ρ* per arm; mean *ρ*_2_ = 0.89 [*t*(44) = 191, adjusted (adj.) *P* < 1.1 × 10^−65^], mean *ρ*_3_ = 0.80 [*t*(44) = 106, adj. *P* < 1.2 × 10^−54^], mean *ρ_X_* = 0.92 [*t*(44) = 210, adj. *P* < 6.3 × 10^−67^]). Such high correlations are not observed among replicate populations in neutral simulations (*t*-test on pairwise *ρ* per arm; mean *ρ*_2_ = −0.03 [*t*(44) = −0.8, adj. *P* > 0.93], mean *ρ*_3_ = 0 [*t*(44) = 0.2, adj. *P* > 0.93], mean *ρ_X_* = 0 [*t*(44) = −0.08, adj. *P* > 0.93]). We visualized the difference between the empirical and simulated replicates by projecting the pairwise correlation matrix in a two-dimensional MDS plot (fig. 1*B*), which highlights the similarity between the empirical replicates for each major chromosome, whereas in the neutral simulations no clustering of replicates was apparent.

Although it is difficult to provide a statistically sound estimate of the number of selection targets, a closer inspection of the distribution of AFCs along the chromosomes indicates that each chromosome harbors multiple selection targets. Based on a single replicate, it is not possible to distinguish whether the ruggedness of the AFCs along the chromosomes is the result of stochastic (recombination and drift) or deterministic forces. Because many of the hills and valleys are well-supported by the replicates, our data indicate that each chromosome arm harbors several distinct loci contributing to temperature adaptation, some of them with opposite effects responsible for “hill-valley-like” pattern. We demonstrated the presence of multiple selection targets on a single chromosome arm for the X chromosome. Computer simulations of a single selection target nicely matched the frequency increase in the target region, but the AFCs for the rest of the chromosome did not fit. We successively added selected alleles with opposite effect (either Samarkand or Oregon-R was favored). This increased the fit between simulated and empirical trajectories for up to six selection targets (supplementary fig. S4, Supplementary Material online) as measured by squared estimate of error (SSE). Additionally, only for the six-target scenario, we did not detect a significant difference between the smoothed empirical and simulated allele frequencies (*P* values in supplementary fig. S4, Supplementary Material online).

Independently of the actual number of selected loci, it is apparent that reducing the genetic variation to two genotypes still leaves a considerable reservoir of favorably selected alleles. This strong selection response is also reflected in effective population size (*N_e_*) estimates based on AFCs. For the X chromosome, *N_e_* barely reaches 25 with a median of 21 and is also rather small on the autosomes (median of 57, supplementary table S2, Supplementary Material online), given a census size of 1,500 flies in each replicate. The effective population size on the X chromosome is much lower than the expected 3/4 reduction relative to the autosomes (Charlesworth 2009). This implies that the efficacy of selection differed between the autosomes and X and that selection was considerably stronger on the X chromosome (see Discussion for possible explanations).

**Fig. 2.**
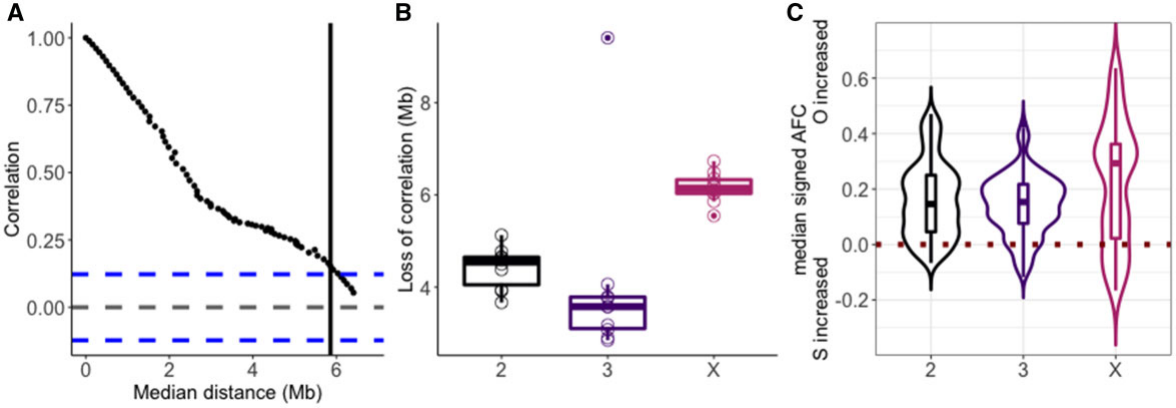
Quantification of the evolutionary response at F20. (*A*) and (*B*) Loss of correlation at the major chromosomes. (*A*) Example of a scatterplot of *ρ* Spearman correlation against the median distance between two windows measured in Mb. The blue dotted lines represent ±1.96/√*m*, with *m* number of windows. (*B*) Jittered boxplots of physical distance in Mb where linkage equilibrium (LE) is reached at a 5% threshold (vertical black line in *A*). (*C*) Boxplots overlaid with violin plots of AFC. A positive (negative) allele frequency change (AFC) indicates that the O genotype increases (decreases) in the window relative to the starting frequency of 0.3. The horizontal dark red dashed line indicates no change in frequency after 20 generations.

The experiment started from two genotypes and in 20 generations the number of recombination events that can uncouple contiguous blocks of Oregon-R/Samarkand alleles which experience a strong frequency increase is limited (in particular as *D. melanogaster* males do not recombine). In the absence of haplotype data from the evolved flies, we used the loss of autocorrelation in AF as a proxy for the decay of LD to quantify the association between genomic sites (fig. 2*A*). The correlation between increasingly distant windows decayed faster on the autosomes (with a median of 4.5 and 3.6 Mb over the 10 replicates for chromosomes 2 and 3) compared with the X chromosome (median of 6.1 Mb) (fig. 2*B*), implying less LD on the autosomes. We attribute the independence of neighboring windows at a lower distance on the autosomes (correlation outside 95% confidence interval) to differences in selection intensities: stronger selection reduces the effective population sizes beyond the 3/4 expected from the ratio of X chromosomes to autosomes, which results in less opportunity for recombination on the X chromosome. Note that the absence of recombination in males makes further strengthens our conclusion as the X-chromosome has more opportunity for recombination than autosomes.

At 29 °C, the two separated parental lines suffered similarly from the high temperature regime and produced low numbers of offspring. When the two strains were combined in the experimental evolution cage, the Oregon-R alleles clearly outcompeted the Samarkand genotypes (figs. 1*A* and 2*C*): the median Oregon-R AFC was significantly higher than 0 (0.15, 0.15, 0.29 for chromosomes 2, 3, and X; adj. *P* < 3.0 × 10^−98^, adj. *P* < 2.0 × 10^−120^, adj. *P* < 3.0 × 10^−19^ on each sign test; fig. 2*C*). Although some heterogeneity can be observed along the chromosome arms (fig. 1*A*; median CV is 0.10, 0.11, 0.18 for chromosomes 2, 3, and X), the median Oregon-R AF increased on each chromosome, ranging from 40% to 65%, which suggests a genome-wide rather than an isolated footprint of selection.

### Exceptionally Strong, Genome-Wide Selection Signatures

**Fig. 3.**
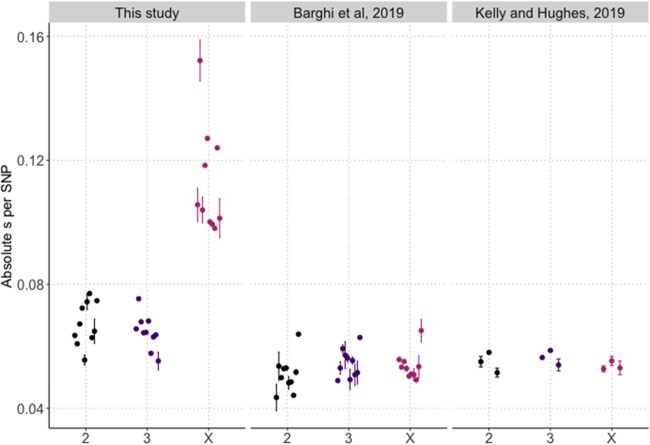
Median absolute selection coefficients (*s*) per SNP across empirical E&R studies for chromosomes 2, 3, and X. The jackknife estimates of the median absolute *s* (dot) are represented along with their 95% confidence intervals (segments). Averaged across the replicates, the effective population size estimates of the autosomes (X) were 55 (20), 228 (203), and 379 (391) for the three studies (this study, Barghi et al. [2019], and Kelly and Hughes [2019], respectively).

With all alleles occurring at similar frequency throughout the entire genome, the comparison of AFCs provides a direct readout of the selective force operating on each SNP—either directly or through linkage to selection targets. To compare the selection experienced in this two-genotype experiment to two other short-term *Drosophila* E&R studies (table 1) that differ in the number of founder genotypes (>800) and consequently in the distribution of starting allele frequencies, we transformed the AFCs into selection coefficients, *s*, which allows the comparison of alleles with different starting frequencies. The pronounced differences in median absolute *s* between the X chromosome and autosomes were specific to the two-genotype experiment (fig. 3). Across all chromosomes, the median absolute *s* was significantly higher for this study compared with the two other studies (fig. 3). This clearly indicates that the two E&R studies with many founder genotypes experienced less selection, not only on the X chromosome, but genome-wide. The differences in selection intensity between the two-genotype experiment and E&R studies with many founder genotypes are also reflected in effective population size (*N_e_*) estimates. With *N_e_* estimates not higher than 61 and 25 for the autosomes and X in all replicates (supplementary table S2, Supplementary Material online), *N_e_* of this study was considerably lower than for the two other E&R studies (see fig. 3 legend), suggesting that a much larger fraction of the genome experienced drastic AFCs. The stronger selection observed in our study may reflect the higher temperature (29 °C rather than average 23 °C in Barghi et al. 2019 and constant 25 °C in Kelly and Hughes 2019). Another explanation for the different selection intensities is that selection is more efficient with two rather than many founder genotypes and leads to more pronounced AFCs. For a highly polygenic trait, an increasing number of founder genotypes will result in more contributing loci, resulting in smaller fitness differences between genotypes.

## Discussion

The idea to start an experimental evolution study with only two genotypes is radically different from current E&R designs, but has already been used before in experimental evolution (e.g., Barnes 1968; Kearsey and Barnes 1970; Nuzhdin et al. 1998). Although strong phenotypic responses were observed in these studies, the small population sizes used, for example in the mouse selection experiments, can result in considerable genetic heterogeneity among replicates which limits the power to detect loci with small/moderate effects. An interesting modification of the two genotype design has been used in fruit flies. From a polymorphic population, two haplotype classes were identified with moderate number of linked allozyme markers, but each haplotype class harbored considerable variation which was not surveyed (Clegg et al. 1976). Evolving populations founded by these two haplotype classes showed very strong selection signatures, but the genomic response between the replicates was heterogeneous, which was attributed to genetic heterogeneity at the unmonitored part of the haplotype classes (Clegg et al. 1976). Overall, previous two-genotype experimental evolution studies were primarily designed to study the phenotypic response, but not to obtain highly parallel genomic selection signatures among replicate populations.

In contrast, this study obtained a highly parallel selection signature which can be attributed to the use of two high frequency genotypes in the founder populations in combination with large census sizes (1,500 individuals). Such a highly parallel selection response provides an excellent tool to study adaptation because selection responses can be reliably distinguished from stochastic patterns—even with a small number of replicates. Consistent with our data, computer simulations showed that two-haplotype E&R studies can be used to experimentally confirm candidate alleles that were previously identified (Langmüller et al. 2021)—similar to a previous secondary E&R experiment (Burny et al. 2020). One further advantage of the highly parallel selection signature seen in this two-haplotype E&R study is that it offers the opportunity to explore epistatic interactions when only a small number of loci are selected. Crossing one inbred strain to at least two other inbred strains (in separate pairwise crosses) provides an excellent system to study epistasis by contrasting the selection response of a candidate locus in different genomic backgrounds. The highly parallel response provides sufficient power to detect even small differences, that is, changes in frequency of the same selection target, due to the genetic background.

Despite the moderate number of generations, the highly parallel selection signature across replicates provides solid support for multiple distinct selection targets on each chromosome arm. First, the rugged AFCs along the chromosomes are concordant across replicates, ruling out major contributions from stochastic forces in the observed topography. Second, consecutive windows of similar AFCs cluster in hills and valleys of variable widths. We showed that computer simulations with six selection targets on the X chromosome provided a better match to the empirical AF patterns than simulations with fewer loci (supplementary fig. S4, Supplementary Material online). Apart from this characterization of selection targets, our data suggest that the number of selection targets may be even larger. We find that hills/valleys of similar absolute AFCs (i.e., similar net selection) are having a different shape (the broadness differs) even when adjusted for spatially variable recombination rates. For example, the selection signature at position 50.7 Mb on chromosome 3 (marked by an arrow in fig. 1*A*;supplementary fig. S3, Supplementary Material online) is much more peaked than other regions. This suggests that despite a moderate number of recombination events during 20 generations, it is possible to obtain a more localized selection signature at this position than at other parts of the genome. This raises the question why other genomic regions are not showing a similar level of resolution as this genomic region. Although it may be possible that recombination rates were not well estimated, we consider this an unlikely explanation given the high quality of recombination map in *D. melanogaster* (Comeron et al. 2012). Alternatively, it may be possible that broad selection signatures are caused by more than one selection target in this region on one of the founder genotypes: If recombination uncouples selection targets (with effects in the same direction), recombined haplotypes are disfavored. We propose that the broad peaks seen in our study are not only the result of limited recombination opportunity, but can be caused by neighboring loci, which are selected in the same direction. This implies that the two genotypes harbor more selection targets than apparent from the selection landscape of hills and valleys (fig. 1).

For a highly polygenic architecture, the selection response of a two-haplotype E&R reflects the net effect of multiple contributing alleles, possibly of different sign, within a selected haplotype block. A similar scenario has been modeled where an admixed genotype is broken up into haplotype blocks, which introgressed when the net effect of all loci within the haplotype block was positive (Sachdeva and Barton 2018). If our two-genotype experiment is extended for more generations, the high parallelism of this setup can be used to study the breaking of the haplotype blocks containing multiple selection targets, by stochastic recombination. This has been done in a recent E&R study in budding yeast, which also started from two inbred founder genotypes, but with a much larger population size and for 960 generations (Kosheleva and Desai 2018). Consistent with a highly polygenic architecture, the fitness of sexual populations continuously increased throughout the entire experiment, possibly by the creation of favorable allelic combinations during the experiment (Hickey and Golding 2018). More generations are needed for this *Drosophila* experiment to determine whether fitness continues to increase as in the yeast study or plateaus when the trait optimum is reached (Franssen et al. 2017; Höllinger et al. 2019).

The classic E&R design maximizing the number of founder genotypes is an excellent approach to identify causative variants for traits with a simple genetic basis (such as C-virus resistance [Martins et al. 2014]), which contributing loci segregate at intermediate frequency in the founder population. Causative variants with a low starting frequency occurring on rare haplotypes are difficult to map because they will result in the increase of a large genomic region (Franssen et al. 2017; Barghi et al. 2019). Computer simulations suggested that a secondary E&R based on multiple replicates of two genotypes can identify causative SNPs when performed for 60 generations at large population sizes (Langmüller et al. 2021). Although large-scale QTL crosses of two genotypes are another powerful approach to identify causative variants, we raise caution that this does not only involve a substantial phenotyping load, but also requires a priori information about the selected phenotype. Although fitness components can also be used for QTL mapping, it is well-understood that different fitness components can provide inconsistent results (e.g., Flatt 2020). E&R, in contrast, does not require information about the selected phenotype and total fitness is measured. Furthermore, phenotyping of outbred flies is restricted to a single measurement for each genotype, which implies that experimental noise reduces the power of QTL crosses. In laboratory natural selection E&R studies, fitness of individuals is not being measured, but it is evaluated across multiple generations as integral part of the experimental design, which provides more reliable results. Consistent with these considerations for simple traits, a simulation study showed that E&R is more powerful than GWAS to identify contributing alleles of a polygenic trait (Vlachos and Kofler 2019). Nevertheless, we are still lacking empirical studies comparing the power of QTL crosses with E&R.

Strong selection responses in populations derived from two founder genotypes imply that one allele provides an advantage relative to the other. Although it is tempting to speculate that the fitness advantage is related to the temperature stress imposed during the experiment, we cannot rule out that the selection response is caused by deleterious alleles that were acquired during the long-term maintenance, since Samarkand and Oregon-R isofemale lines have been collected more than 90 years ago (Lindsley and Grell 1968). Isofemale lines are typically maintained at small population sizes, which renders most mutations effectively neutral (Ohta 1973; Kimura 1983) and could lead to the accumulation of deleterious alleles that are fixed in the parental strains. Consistent with the presence of deleterious alleles, we noticed that heterozygous F1 flies produced a larger number of eggs at 29 °C than the inbred strains which had difficulties to sustain the next generation. If deleterious alleles are the primary driver of the observed AFCs, the predominant increase of Oregon-R alleles would suggest that Samarkand has accumulated more deleterious alleles than Oregon-R. This conclusion is not supported by obvious fitness differences of the two parental genotypes at 29 °C. Alternatively, the lack of clear fitness differences in the parental lines could be explained by overdominance, but the reason for the predominant frequency increase of Oregon-R allele frequencies remains unclear. Additional generations at 29 °C would help to distinguish between both explanations. Deleterious alleles would be ultimately purged while overdominance would result in a stable equilibrium frequency. A third interpretation of the data is based on epistatic interactions between Samarkand and Oregon-R alleles. If a few Samarkand alleles interact with many Oregon-R alleles, this could account for the advantage of heterozygotes and the predominance of Oregon-R alleles among the selectively favored ones. Epistatic interactions could be further tested when the Oregon-R genotype is competed with other (inbred) genotypes in separate pairwise competition experiments.

A particularly interesting result was the different selection signature on the X chromosome compared with the autosomes. More pronounced AFCs, and hence higher selection coefficients, were found on the X chromosome translating in lower *N_e_* estimate than expected, that is, lower than ¾ of the *N_e_* on the autosomes. We propose two not mutually exclusive explanations for this observation: 1) the selected loci may be (partially) recessive which allows for a more efficient selection on the X chromosome (Charlesworth et al. 1987; Mank et al. 2010; Meisel and Connallon 2013); 2) the X chromosome has either more contributing loci or they may have larger effects. Although it is hard to hypothesize about the distribution (number and location) of the selection targets after only 20 generations, we favor the dominance explanation because it is not apparent why the number of selection targets or their effect sizes should be different between the X chromosome and the autosomes.

## Supplementary Material

Supplementary data are available at *Genome Biology and Evolution* online.

## Supplementary Material

evab239_Supplementary_Data

## References

[evab239-B1] Angilletta MJ Jr. 2009. Thermal adaptation: a theoretical and empirical synthesis. Oxford, United Kingdom: Oxford University Press.

[evab239-B2] Aphalo PJ. 2021. ggpmisc: miscellaneous extensions to ’ggplot2’. Available from: https://docs.r4photobiology.info/ggpmisc/, https://github.com/aphalo/ggpmisc.

[evab239-B3] Baldwin-Brown JG , Long AD, Thornton KR. 2014. The power to detect quantitative trait loci using resequenced, experimentally evolved populations of diploid, sexual organisms. Mol Biol Evol. 31(4):1040–1055. 24441104 10.1093/molbev/msu048 PMC3969567

[evab239-B4] Barghi N , Hermisson J, Schlötterer C. 2020. Polygenic adaptation: a unifying framework to understand positive selection. Nat Rev Genet. 21(12):769–781. 32601318 10.1038/s41576-020-0250-z

[evab239-B6] Barghi N , et al 2019. Genetic redundancy fuels polygenic adaptation in *Drosophila*. PLoS Biol. 17(2):e3000128. 30716062 10.1371/journal.pbio.3000128 PMC6375663

[evab239-B7] Barnes BW. 1968. Stabilising selection in *Drosophila melanogaster*. Heredity 23(3):433–442. 5250124 10.1038/hdy.1968.54

[evab239-B8] Barton NH. 1995. A general model for the evolution of recombination. Genet Res. 65(2):123–144. 7605514 10.1017/s0016672300033140

[evab239-B9] Barton NH , Etheridge AM. 2018. Establishment in a new habitat by polygenic adaptation. Theor Popul Biol. 122:110–127. 29246460 10.1016/j.tpb.2017.11.007

[evab239-B10] Barton N , Hermisson J, Nordborg M. 2019. Why structure matters. eLife 8:e45380. 30895925 10.7554/eLife.45380 PMC6428565

[evab239-B11] Berg JJ , Coop G. 2014. A population genetic signal of polygenic adaptation. PLOS Genet. 10(8):e1004412. 25102153 10.1371/journal.pgen.1004412 PMC4125079

[evab239-B12] Berg JJ , et al 2019. Reduced signal for polygenic adaptation of height in UK Biobank. eLife. 8:e39725. 30895923 10.7554/eLife.39725 PMC6428572

[evab239-B13] Bolnick DI , Barrett RDH, Oke KB, Rennison DJ, Stuart YE. 2018. Non parallel evolution. Annu Rev Ecol Evol Syst. 49(1):303–330.

[evab239-B15] Burny C , Nolte V, Nouhaud P, Dolezal M, Schlötterer C. 2020. Secondary evolve and resequencing: an experimental confirmation of putative selection targets without phenotyping. Genome Biol Evol. 12(3):151–159. 10.1093/gbe/evaa036 PMC7144549 32159748

[evab239-B17] Charlesworth B. 2009. Effective population size and patterns of molecular evolution and variation. Nat Rev Genet. 10(3):195–205. 19204717 10.1038/nrg2526

[evab239-B18] Charlesworth B , Coyne JA, Barton NH. 1987. The relative rates of evolution of sex chromosomes and autosomes. Am Nat. 130(1):113–146.

[evab239-B19] Chen J , Nolte V, Schlötterer C. 2015. Temperature stress mediates decanalization and dominance of gene expression in *Drosophila melanogaster*. PLoS Genet. 11(2):e1004883. 25719753 10.1371/journal.pgen.1004883 PMC4342254

[evab239-B20] Clegg MT , Kidwell JF, Kidwell MG, Daniel NJ. 1976. Dynamics of correlated genetic systems. I. Selection in the region of the glued locus of *Drosophila melanogaster*. Genetics 83(4):793. 823074 10.1093/genetics/83.4.793 PMC1213552

[evab239-B21] Comeron JM , Ratnappan R, Bailin S. 2012. The many landscapes of recombination in *Drosophila melanogaster*. PLoS Genet. 8(10):e1002905. 23071443 10.1371/journal.pgen.1002905 PMC3469467

[evab239-B22] Danecek P , et al 2011. The variant call format and VCFtools. Bioinformatics 27(15):2156–2158. 21653522 10.1093/bioinformatics/btr330 PMC3137218

[evab239-B23] Dayan DI , et al 2019. Population genomics of rapid evolution in natural populations: polygenic selection in response to power station thermal effluents. BMC Evol Biol. 19(1):61. 30808292 10.1186/s12862-019-1392-5 PMC6390305

[evab239-B24] Efron B , Tibshirani RJ. 1994. An introduction to the bootstrap. New York: CRC Press.

[evab239-B25] Ehrenreich IM , et al 2010. Dissection of genetically complex traits with extremely large pools of yeast segregants. Nature 464(7291):1039–1042. 20393561 10.1038/nature08923 PMC2862354

[evab239-B26] Felsenstein J. 1974. The evolutionary advantage of recombination. Genetics 78(2):737–737. 4448362 10.1093/genetics/78.2.737 PMC1213231

[evab239-B27] Flatt T. 2020. Life-history evolution and the genetics of fitness components in *Drosophila melanogaster*. Genetics 214(1):3–48. 31907300 10.1534/genetics.119.300160 PMC6944413

[evab239-B28] Franssen S , Kofler R, Schlötterer C. 2017. Uncovering the genetic signature of quantitative trait evolution with replicated time series data. Heredity 118(1):42–51. 27848948 10.1038/hdy.2016.98 PMC5176121

[evab239-B30] Garrison E , Marth G. 2012. Haplotype-based variant detection from short-read sequencing. arXiv. 1207.3907.

[evab239-B31] Garland T , Rose MR. 2009. Experimental evolution: concepts, methods, and applications of selection experiments. Berkeley (CA): University of California Press.

[evab239-B32] Gatti DM , et al 2014. Quantitative trait locus mapping methods for diversity outbred mice. Genes Genom Genet. 4(9):1623–1633. 10.1534/g3.114.013748 PMC4169154 25237114

[evab239-B33] Gómez-Sánchez D , Schlötterer C. 2018. ReadTools: a universal toolkit for handling sequence data from different sequencing platforms. Mol Ecol Resour. 18(3):676–680. 29171165 10.1111/1755-0998.12741

[evab239-B34] Gower JC. 1966. Some distance properties of latent root and vector methods used in multivariate analysis. Biometrika 53(3–4):325–338.

[evab239-B36] Griffin PC , Hangartner SB, Fournier-Level A, Hoffmann AA. 2017. Genomic trajectories to desiccation resistance: convergence and divergence among replicate selected Drosophila lines. Genetics 205(2):871–871. 28007884 10.1534/genetics.116.187104 PMC5289857

[evab239-B37] Knaus BJ , Grünwald NJ. 2017. vcfr: a package to manipulate and visualize variant call format data in R. Mol Ecol Resour. 17(1):44–53. 27401132 10.1111/1755-0998.12549

[evab239-B38] Hardy CM , et al 2018. Genome-wide analysis of starvation-selected *Drosophila melanogaster*—a genetic model of obesity. Mol Biol Evol. 35(1):50–65. 29309688 10.1093/molbev/msx254 PMC5850753

[evab239-B42] Herrmann M , Yampolsky LY. 2021. False and true positives in arthropod thermal adaptation candidate gene lists. Genetica 149(3):143–153. 33963492 10.1007/s10709-021-00122-w

[evab239-B43] Hickey DA , Golding GB. 2018. The advantage of recombination when selection is acting at many genetic loci. J Theor Biol. 442:123–128. 29355539 10.1016/j.jtbi.2018.01.018

[evab239-B44] Hill WG , Robertson A. 1968. Linkage disequilibrium in finite populations. Theor Appl Genet. 38(6):226–231. 24442307 10.1007/BF01245622

[evab239-B45] Hoffmann AA. 2010. Physiological climatic limits in *Drosophila*: patterns and implications. J Exp Biol. 213(6):870. 20190112 10.1242/jeb.037630

[evab239-B46] Hoffmann AA , Sørensen JG, Loeschcke V. 2003. Adaptation of *Drosophila* to temperature extremes: bringing together quantitative and molecular approaches. J Therm Biol. 28(3):175–216.

[evab239-B47] Höllinger I , Pennings PS, Hermisson J. 2019. Polygenic adaptation: from sweeps to subtle frequency shifts. PLoS Genet. 15(3):e1008035. 30893299 10.1371/journal.pgen.1008035 PMC6443195

[evab239-B48] Kapun M , van Schalkwyk H, McAllister B, Flatt T, Schlötterer C. 2014. Inference of chromosomal inversion dynamics from Pool-Seq data in natural and laboratory populations of *Drosophila melanogaster*. Mol Ecol. 23(7):1813–1827. 24372777 10.1111/mec.12594 PMC4359753

[evab239-B49] Kearsey MU , Barnes BW. 1970. Variation for metrical characters in *Drosophila* populations. II. Natural selection. Heredity 25(1):11–21. 5004595 10.1038/hdy.1970.2

[evab239-B51] Kelly JK , Hughes KA. 2019. Pervasive linked selection and intermediate-frequency alleles are implicated in an evolve-and-resequencing experiment of *Drosophila simulans*. Genetics 211(3):943–961. 30593495 10.1534/genetics.118.301824 PMC6404262

[evab239-B52] Kessner D , Novembre J. 2015. Power analysis of artificial selection experiments using efficient whole genome simulation of quantitative traits. Genetics 199(4):991. 25672748 10.1534/genetics.115.175075 PMC4391575

[evab239-B53] Kimura M. 1983. Rare variant alleles in the light of the neutral theory. Mol Biol Evol. 1(1):84–93. 6599962 10.1093/oxfordjournals.molbev.a040305

[evab239-B54] Kofler R , Pandey RV, Schlötterer C. 2011. PoPoolation2: identifying differentiation between populations using sequencing of pooled DNA samples (Pool-Seq). Bioinformatics 27(24):3435–3436. 22025480 10.1093/bioinformatics/btr589 PMC3232374

[evab239-B55] Kofler R , Schlötterer C. 2014. A guide for the design of evolve and resequencing studies. Mol Biol Evol. 31(2):474–483. 24214537 10.1093/molbev/mst221 PMC3907048

[evab239-B56] Königer A , Arif S, Grath S. 2019. Three quantitative trait loci explain more than 60% of variation for chill coma recovery time in a natural population of *Drosophila ananassae*. Genes Genom Genet. 9(11):3715–3725. 10.1534/g3.119.400453 PMC6829138 31690597

[evab239-B57] Kosheleva K , Desai MM. 2018. Recombination alters the dynamics of adaptation on standing variation in laboratory yeast populations. Mol Biol Evol. 35(1):180–201. 29069452 10.1093/molbev/msx278 PMC5850740

[evab239-B58] Langmüller AM , Dolezal M, Schlötterer C. 2021. Fine mapping without phenotyping: identification of selection targets in secondary evolve and resequence experiments. Genome Biol Evol. 13(8):evab154. 34190980 10.1093/gbe/evab154 PMC8358229

[evab239-B59] Langmüller AM , Schlötterer C. 2020. Low concordance of short-term and long-term selection responses in experimental Drosophila populations. Mol Ecol. 29(18):3466–3475. 32762052 10.1111/mec.15579 PMC7540288

[evab239-B60] Leisch F , Kostyshack MS, T.R.U.E. LazyData. 2019. Package ’bootstrap’. Diabete 14:1.

[evab239-B61] Láruson ÁJ , Yeaman S, Lotterhos KE. 2020. The importance of genetic redundancy in evolution. Trends Ecol Evol. 35(9):809–822. 32439075 10.1016/j.tree.2020.04.009

[evab239-B62] Li H. 2011. A statistical framework for SNP calling, mutation discovery, association mapping and population genetical parameter estimation from sequencing data. Bioinformatics 27(21):2987–2993. 21903627 10.1093/bioinformatics/btr509 PMC3198575

[evab239-B63] Li H , et al 2009. The sequence alignment/map format and SAMtools. Bioinformatics 25(16):2078–2079. 19505943 10.1093/bioinformatics/btp352 PMC2723002

[evab239-B64] Lindsley DL , Grell EH. 1968. Genetic variations of *Drosophila melanogaster*. Washington (DC): Carnegie Institute of Washington.

[evab239-B65] Long A , Liti G, Luptak A, Tenaillon O. 2015. Elucidating the molecular architecture of adaptation via evolve and resequence experiments. Nat Rev Genet. 16(10):567–582. 26347030 10.1038/nrg3937 PMC4733663

[evab239-B66] Lou RN , Therkildsen NO, Messer PW. 2020. The effects of quantitative trait architecture on detection power in short-term artificial selection experiments. Genes Genom Genet. 10(9):3213–3213. 10.1534/g3.120.401287 PMC7466968 32646912

[evab239-B67] Mank JE , Vicoso B, Berlin S, Charlesworth B. 2010. Effective population size and the Faster-X effect: empirical results and their interpretation. Evolution 64(3):663–674. 19796145 10.1111/j.1558-5646.2009.00853.x

[evab239-B68] Martins NE , et al 2014. Host adaptation to viruses relies on few genes with different cross-resistance properties. Proc Natl Acad Sci U S A. 111(16):5938. 24711428 10.1073/pnas.1400378111 PMC4000853

[evab239-B71] Meisel RP , Connallon T. 2013. The faster-X effect: integrating theory and data. Trends Genet. 29(9):537–544. 23790324 10.1016/j.tig.2013.05.009 PMC3755111

[evab239-B1278440] Miller SA, , Dykes DD, , Polesky HF. 1988. A simple salting out procedure for extracting DNA from human nucleated cells. Nucleic Acids Res. 16(3):1215. 3344216 10.1093/nar/16.3.1215 PMC334765

[evab239-B73] Nené NR , Dunham AS, Illingworth CJR. 2018. Inferring fitness effects from time-resolved sequence data with a delay-deterministic model. Genetics 209(1):255–264. 29500183 10.1534/genetics.118.300790 PMC5937181

[evab239-B75] Nuzhdin SV , Keightley PD, Pasyukova EG, Morozova EA. 1998. Mapping quantitative trait loci affecting sternopleural bristle number in Drosophila melanogaster using changes of marker allele frequencies in divergently selected lines. Genet Res. 72(2):79–91. 9883095 10.1017/s001667239800336x

[evab239-B76] Ohta T. 1973. Slightly deleterious mutant substitutions in evolution. Nature 246(5428):96–98. 4585855 10.1038/246096a0

[evab239-B78] Otto SP , Lenormand T. 2002. Resolving the paradox of sex and recombination. Nat Rev Genet. 3(4):252–261. 11967550 10.1038/nrg761

[evab239-B80] Pritchard JK , Di Rienzo A. 2010. Adaptation – not by sweeps alone. Nat Rev Genet. 11(10):665–667. 20838407 10.1038/nrg2880 PMC4652788

[evab239-B81] Pritchard JK , Pickrell JK, Coop G. 2010. The genetics of human adaptation: hard sweeps, soft sweeps, and polygenic adaptation. Curr Biol. 20(4):R208–R215. 20178769 10.1016/j.cub.2009.11.055 PMC2994553

[evab239-B82] Quinlan AR , Hall IM. 2010. BEDTools: a flexible suite of utilities for comparing genomic features. Bioinformatics 26(6):841–842. 20110278 10.1093/bioinformatics/btq033 PMC2832824

[evab239-B83] R Core Team. 2018. R: a language and environment for statistical computing. R Foundation for Statistical Computing Vienna, Austria.

[evab239-B84] Rêgo A , Messina FJ, Gompert Z. 2019. Dynamics of genomic change during evolutionary rescue in the seed beetle *Callosobruchus maculatus*. Mol Ecol. 28(9):2136–2154. 30963641 10.1111/mec.15085

[evab239-B85] Roze D , Barton NH. 2006. The Hill–Robertson effect and the evolution of recombination. Genetics 173(3):1793. 16702422 10.1534/genetics.106.058586 PMC1526660

[evab239-B86] Sachdeva H , Barton NH. 2018. Introgression of a block of genome under infinitesimal selection. Genetics 209(4):1279–1303. 29895560 10.1534/genetics.118.301018 PMC6063232

[evab239-B87] Schlötterer C , Kofler R, Versace E, Tobler R, Franssen SU. 2015. Combining experimental evolution with next-generation sequencing: a powerful tool to study adaptation from standing genetic variation. Heredity 114(5):431–440. 25269380 10.1038/hdy.2014.86 PMC4815507

[evab239-B88] Schlötterer C , Tobler R, Kofler R, Nolte V. 2014. Sequencing pools of individuals—mining genome-wide polymorphism data without big funding. Nat Rev Genet. 15(11):749–763. 25246196 10.1038/nrg3803

[evab239-B89] Seabra SG , et al 2018. Different genomic changes underlie adaptive evolution in populations of contrasting history. Mol Biol Evol. 35(3):549–563. 29029198 10.1093/molbev/msx247

[evab239-B91] Sella G , Barton NH. 2019. Thinking about the evolution of complex traits in the era of genome-wide association studies. Annu Rev Genomics Hum Genet. 20(1):461–493. 31283361 10.1146/annurev-genom-083115-022316

[evab239-B92] Sohail M , et al 2019. Polygenic adaptation on height is overestimated due to uncorrected stratification in genome-wide association studies. eLife 8:e39702. 30895926 10.7554/eLife.39702 PMC6428571

[evab239-B93] Tan A , Abecasis GR, Kang HM. 2015. Unified representation of genetic variants. Bioinformatics 31(13):2202. 25701572 10.1093/bioinformatics/btv112 PMC4481842

[evab239-B94] Taus T , Futschik A, Schlötterer C. 2017. Quantifying selection with pool-seq time series data. Mol Biol Evol. 34(11):3023–3034. 28961717 10.1093/molbev/msx225 PMC5850601

[evab239-B95] Thurmond J , et al 2019. FlyBase 2.0: the next generation. Nucleic Acids Res. 47(D1):D759–D765. 30364959 10.1093/nar/gky1003 PMC6323960

[evab239-B96] Turchin MC , et al 2012. Evidence of widespread selection on standing variation in Europe at height-associated SNPs. Nat Genet. 44(9):1015–1019. 22902787 10.1038/ng.2368 PMC3480734

[evab239-B97] Turner TL , Stewart AD, Fields AT, Rice WR, Tarone AM. 2011. Population-based resequencing of experimentally evolved populations reveals the genetic basis of body size variation in *Drosophila melanogaster*. PLoS Genet. 7(3):e1001336. 21437274 10.1371/journal.pgen.1001336 PMC3060078

[evab239-B98] Vlachos C , et al 2019. Benchmarking software tools for detecting and quantifying selection in evolve and resequencing studies. Genome Biol. 20(1):169–169. 31416462 10.1186/s13059-019-1770-8 PMC6694636

[evab239-B99] Vlachos C , Kofler R. 2018. MimicrEE2: genome-wide forward simulations of Evolve and Resequencing studies. PLoS Comput Biol. 14(8):e1006413. 30114186 10.1371/journal.pcbi.1006413 PMC6112681

[evab239-B100] Vlachos C , Kofler R. 2019. Optimizing the power to identify the genetic basis of complex traits with evolve and resequence studies. Mol Biol Evol. 36(12):2890–2905. 31400203 10.1093/molbev/msz183 PMC6878953

[evab239-B101] Wickham H. 2016. ggplot2: elegant graphics for data analysis. New York: Springer.

[evab239-B102] Yeaman S. 2015. Local adaptation by alleles of small effect. Am Nat. 186(S1):S74–S89. 26656219 10.1086/682405

