# Supplementary material for: Highly Parallel Genomic Selection Response in Replicated Drosophila melanogaster Populations with Reduced Genetic Variation
Source: Genome Biol Evol. 2021 Oct 25;13(11):evab239. doi: 10.1093/gbe/evab239 (PMC8599828; doi:10.1093/gbe/evab239)
Supplement: evab239_Supplementary_Data [file evab239_supplementary_data.docx]

**SUPPLEMENTARY INFORMATION**

**Supplementary Table 1.** Establishment of the parental markers catalogue split per arm.

| # SNPs  (# MNPs) | X | 2L | 2R | 3L | 3R | 4 |
| --- | --- | --- | --- | --- | --- | --- |
| raw | 127,479  (12,611) | 161,997  (18,220) | 150,592  (15,566) | 179,744  (19,528) | 178,698  (16,289) | 1,639  (88) |
| decomposed bi-allelic SNPs outside repeats and not within 5bp of an INDEL  (soft filters) | 121,540  (0) | 167,125  (0) | 146,717  (0) | 178,463  (0) | 176,296  (0) | 923  (0) |
| after hard filters | 95,913  *31,391 | 148,293  *47,526 | 128,710  *38,227 | 156,519  *48,872 | 154,063  *49,807 | 567  *493 |
| parental markers | 63,818 | 100,315 | 89,955 | 107,133 | 103,777 | 72 |

*Indicates the number of SNPs that are genotyped 1/1 in both parents and shall not be considered as marker SNPs.

**Supplementary Table 2.** *N_e_* estimates per replicate for the autosomes and X separately.

| **Sample** | ***N*_e_ estimate**  **Autosomes / X** |
| --- | --- |
| R1 | 58 / 22 |
| R2 | 57 / 14 |
| R3 | 53 / 22 |
| R4 | 54 / 19 |
| R5 | 56 / 19 |
| R6 | 50 / 22 |
| R7 | 48 / 25 |
| R8 | 60 / 20 |
| R9 | 59 / 17 |
| R10 | 61 / 23 |


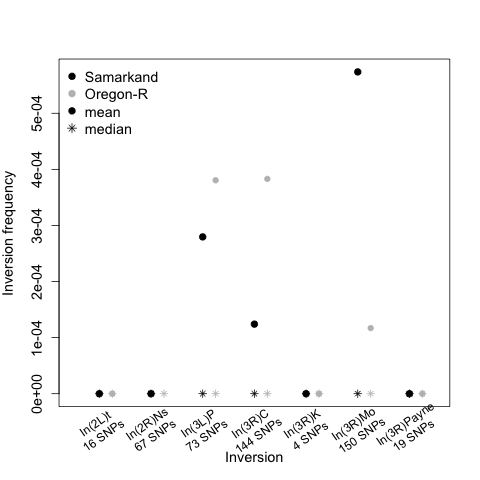


**Supplementary Fig. S1.** Inversion status in Samarkand (black) and Oregon-R (gray) parents of known inversions from Kapun et al, 2014.

The mean and median frequencies of the inversions as well as the number of measurable marker SNPs are reported.


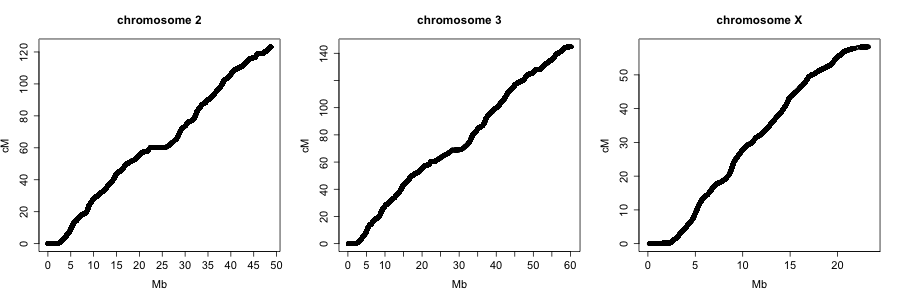


**Supplementary Fig. S2.** Marey maps for the major arms showing the genetic position (y-axis) versus the physical position (x-axis) per parental maker. We clearly see the different recombination domains. We did not represent chromosome 4, lacking crossovers (discussed in Hartmann and Sekelsky, 2017).


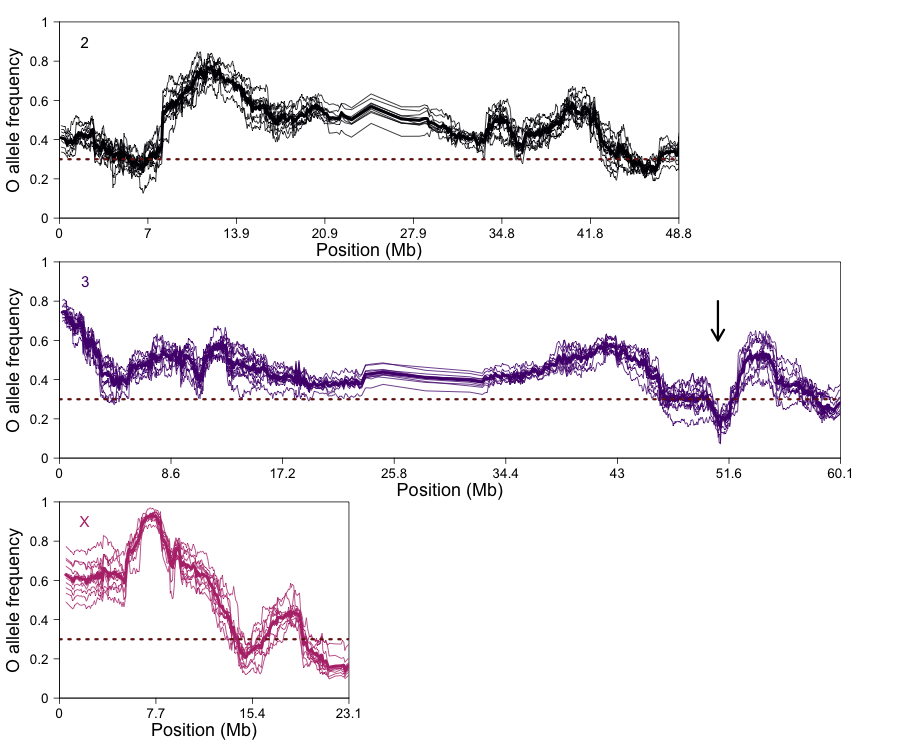


**Supplementary Fig. S3.** From cM to Mb unit. Similar Figure as Fig. 1A but using the Mb unit on the x-axis. The arrows indicates the position of a narrow block on chromosome 3 (50.7Mb, see Discussion).

**
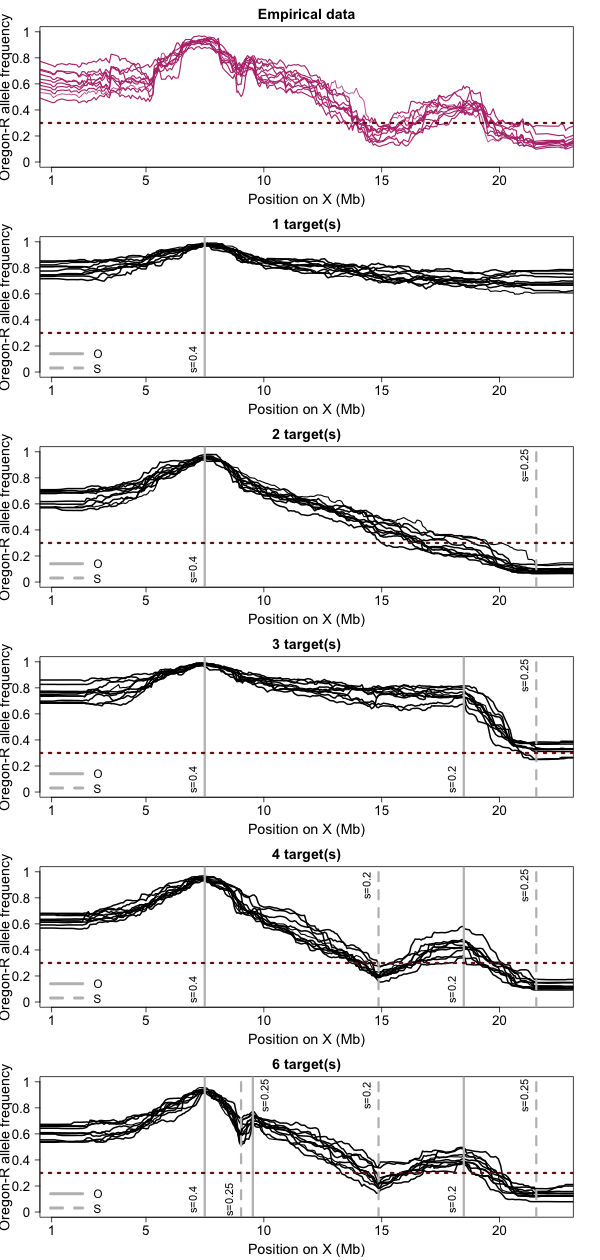
**

**Supplementary Fig. S4.** Empirical Oregon-R allele frequencies (y-axis) on the X chromosome in Mb unit (top) and simulated frequencies obtained with an increasing number of targets (from top to bottom; 1, 2, 3, 4, 6). The gray vertical lines indicate the position of the selected alleles, either Samarkand (plain line) or Oregon-R (dashed line). The selection coefficient value associated to each target is indicated on the graph. One pink (black) line represents one out of ten empirical (simulated) replicates. The horizontal dotted line represents the starting Oregon-R allele frequency (0.3). The sum of squared estimate of error (SSE) decreased with the number of targets (25.41, 2.47, 18.52, 0.73 and 0.35 from 1 to 6 targets respectively). Only for the 6-target scenario the differences between the smoothed empirical and simulated allele frequencies were not significant (t(255)=30.77, adjusted (adj.) p<4.2⨉10^-87^; t(255)=4.23, adj. p<5.4⨉10^-5^; t(255)=29.60, adj. p<4.6⨉10^-84^; t(255)=2.80, adj. p=6.97⨉10^-3^; t(255)=-1.27, adj. p=0.21 from 1 to 6 targets).

**REFERENCES**

Hartmann MA, Sekelsky J. 2017. The absence of crossovers on chromosome 4 in *Drosophila melanogaster*: Imperfection or interesting exception? Fly. 11(4):253–259.
